# Reactivation of Histoplasmosis Disseminated into the Central Nervous System Presenting as a Stroke

**DOI:** 10.7759/cureus.6525

**Published:** 2019-12-31

**Authors:** Linda Klumpp, Gustine Liu-Young, Fagun Modi, Jeffrey Jordan, Rony Shah

**Affiliations:** 1 Internal Medicine, Citrus Memorial Hospital, Inverness, USA; 2 Infectious Disease, Citrus Memorial Hospital, Inverness, USA; 3 Pulmonary/Critical Care, Citrus Memorial Hospital, Inverness, USA

**Keywords:** histoplasmosis, central nervous system (cns), dissemination, stroke, mycosis, amphotericin b, itraconazole, reactivation, histoplasma capsulatum, immunocompetent patient

## Abstract

Histoplasmosis is one of the most prevalent endemic mycosis in the United States. Patients with a previous history of histoplasmosis have a risk of reinfection in the future. Individuals with impaired immunity and those who have massive re-exposure to H. capsulatum, their defenses against this organism can be overwhelmed and diseases can recur. We present a unique case of reactivation disseminated histoplasmosis in an immunocompetent patient.

We present a case of a 75-year-old male who presented to the ER on two separate occasions for stroke-like symptoms with progressive falls, impaired speech, hand tremor, confusion and generalized weakness. CT of the head without contrast on both occasions showed chronic atrophy and microvascular changes but no acute abnormalities. MRI could not be performed due to pacemaker incompatibility. EKG showed paced rhythm. The only abnormal lab was a creatinine level of 1.6. Neurology was consulted and they ordered an EEG and lumbar puncture during his second hospitalization. EEG showed generalized slowing, suggestive of diffuse brain dysfunction. Lumbar puncture showed WBC: 103, protein: 172, lymphocytes: 88%, neutrophils: 11%, monocytes: 1%. Following the lumbar puncture, Infectious Disease was consulted. On further investigation, it was discovered that the patient was previously treated for oral histoplasmosis with itraconazole for three months. Cerebrospinal fluid (CSF) was positive for histoplasmosis antigen titer 1:64. Serology was positive for histoplasmosis antibody complement fixation titer of 1:32. The patient was treated with liposomal amphotericin B for six weeks. With treatment, his serology titers continued to improve. The patient was discharged home on itraconazole 200 mg for lifetime, due to his previous history of oral histoplasmosis. On his three-month follow-up, his serology titer was <1:8.

Histoplasmosis should be considered in the differential diagnosis of patients who present with chronic meningitis, cerebral vascular accident, focal brain or spinal cord lesions, and encephalitis.

## Introduction

Histoplasmosis is the most prevalent endemic mycosis in the United States [1]. Progressive disseminated histoplasmosis occurs in about one in 2,000 patients with acute infection [2]. Patients with a previous history of histoplasmosis have a risk of reinfection in the future. Individuals with impaired immunity and those who have massive re-exposure to Histoplasma capsulatum (*H*. *capsulatum*), their defenses against this organism can be overwhelmed and diseases can recur. We present a unique case of reactivation disseminated histoplasmosis in an immunocompetent patient.

## Case presentation

We present a case of a 75-year-old male who had been residing in Puerto Escondido, Mexico. Our patient has a known history of coronary artery disease, cerebral vascular accident, diabetes mellitus II, chronic kidney disease stage III, hypertension, and pacemaker implantation secondary to sick sinus syndrome. The patient presented to the emergency room (ER) in December 2018, on two occasions within one week with stroke-like symptoms. On initial presentation, in early December the patient presented to the emergency room with complaints of progressive falls, impaired speech, hand tremors and generalized weakness. On physical exam: oral temperature 98.5 F, pulse 68 bpm, respiratory rate 17 pm, blood pressure 159/74 mmHg, oxygen saturation 98% on room air. The patient was ataxic with right-sided weakness. CT scan of the head without contrast did not show any acute abnormalities or hemorrhage, results were consistent with chronic atrophy and microvascular changes. Contrast dye could not be used to due stage III chronic kidney disease. CT of the cervical spine showed stable degenerative disc disease. Unfortunately, MRI could not be performed as his pacemaker was not MRI compatible. EKG rhythm was consistent with atrial and ventricular pacemaker, and no irregularities were recorded. Laboratory values were: WBC 5.4, B12 197, folic acid 7.8, TSH 0.928, creatinine 1.6, and no electrolyte imbalances, hemoglobin A1c 6.1. Lipid panel: Total cholesterol 181, LDL 50, HDL 25. The patient was discharged on aspirin and statin therapy with home healthcare.

Two days later the patient presented to the ER with complaints of ataxia, confusion, dysarthria, and visual impairment. Vital signs were: pulse 71, respiratory rate 15, blood pressure 126/75, oxygen saturation 97% on room air. The patient was ataxic with right-sided visual impairment, confusion and had difficulty speaking. CAT scan of the head again showed no acute abnormality or hemorrhage. Again, MRI could not be performed due to pacemaker incompatibility. EKG was unchanged from previous visit. The differential diagnosis consisted of possible embolic stroke, lacunar infarction, and hypercoagulable state. The patient rapidly worsened with apraxia of gait and sitting. Neurology decided to perform lumbar puncture and obtain an EEG. The EEG showed generalized slowing, suggestive of diffuse brain dysfunction. The EEG did not refute or support epilepsy. A lumbar puncture was performed. Cerebrospinal fluid (CSF): WBC 103, protein 172, lymphocytes 88%, neutrophils 11%, monocytes 1%; venereal disease research laboratory test (VDRL): Non-reactive, lyme negative, and S100 protein negative for malignancy. Infectious Disease was consulted at that time with lab findings. HIV serology was ordered, both HIV 1 p24 antigen and HIV antibody were negative. The patient’s wife mentioned she was familiar with Infectious Disease physician, as she treated her husband 11 months prior for oral Histoplasmosis. In retrospect, the patient had confirmed biopsy of oral Histoplasmosis in January 2018. A three-month course of itraconazole was completed at that time. The patient returned back to Puerto Escondido, Mexico in August 2018 following treatment. In light of patient’s history and clinical findings he was started on liposomal amphotericin 5 mg/kilo/day intravenously, prior to final CSF results. On day three of treatment, the patient had significant mentation improvement. Days later the cerebral spinal fluid results confirmed Histoplasmosis antigen titer 1:64. Serology confirmed Histoplasmosis antibody complement fixation titer of 1:32. Shortly after the initiation of treatment, the patient’s renal function worsened, with an elevated creatinine level of 2.1. Increased IV hydration helped to improve creatinine level to 1.8. Days later, the patient developed shortness of breath and acute hypoxia. At that time, the patient was placed on Bi-Pap. His respiratory symptoms improved and he was able to receive oxygen supplementation via nasal canula. CT of the chest was performed which was positive for pleural effusion and right upper lobe pulmonary nodule measuring 8 mm. Mediastinal lymphadenopathy was also present with largest lymph node measuring 1.2 x 2.0 cm. At that time bronchoscopy +/- endobronchial ultrasound (EBUS) was considered for biopsy of the lymph node and the nodule. Bronchoscopy was postponed due to patient receiving anticoagulation therapy. It was decided at that time a repeat CT of the chest would be performed in 4-6 weeks to monitor the size nodule and pre-tracheal lymphadenopathy. CT with contrast would be completed if renal function was stable. If there was shrinkage these findings can be correlated to Histoplasmosis and successful treatment with Amphotericin B. In early January, blood flow cytometry was performed to rule out Lymphoma. There was no evidence of leukemia or lymphoma. Late January 2019 repeat blood titers improved, with results of Histoplasmosis antibody of 1:16. The patient’s respiratory status continued to improve with diuresis. Creatinine level again worsened and increased to 2.6. Acute kidney injury was secondary to long-term use of Amphotericin B and intermittent furosemide for pleural effusions. Three weeks later our patient was discharged after a six-week course of Liposomal Amphotericin 300 mg IV daily. He will remain on oral itraconazole 200 mg BID for one year and then continue the course of itraconazole 200 mg daily for his lifetime. The most recent Histoplasmosis titer is < 1:8. The patient has continued to improve clinically and remains stable. Improvement can be correlated to prolonged and continued treatment with antifungals. To date the patient is stable without neurological deficit.

## Discussion

The diagnosis of disseminated histoplasmosis of central nervous system (CNS) can be challenging. Multiple concomitant tests are required to establish a diagnosis due to the low sensitivity of diagnostic tests. Even though CSF fungal culture is the most specific test, its sensitivity can be as low as 25% [3]. *H*. *capsulatum* can take up to six weeks to grow in culture, which significantly delays diagnosis and treatment. The specificity of the CSF Histoplasma antigen remains high (96%). Anti-Histoplasma antibodies in the CSF have good sensitivity (80-89%) and specificity (83%) can be a helpful adjunct to establish the diagnosis [3]. Our case demonstrates the importance of clinical diagnosis when physical limitations prevent utilization of medical imaging. It is essential to get an adequate history and physical, including travel history and environmental exposures, when trying to narrow down a differential diagnosis to an infectious process.

There is no definitive treatment for CNS histoplasmosis. The recommended treatment is liposomal amphotericin B (5 mg/kg daily for a total of 175 mg/kg over 4-6 weeks) followed by itraconazole (200 mg 2-3 times daily) for at least one year and until resolution of CSF abnormalities, including negative Histoplasma antigen [3]. Our patient will remain on lifelong itraconazole due to his previous history of oral histoplasmosis.

## Conclusions

CNS involvement occurs in 5 to 20% of cases of disseminated histoplasmosis and appears to be more common in those with underlying immunosuppressive disorders. Histoplasmosis should be considered in the differential diagnosis of patients with chronic meningitis, focal brain or spinal cord lesions, and encephalitis. Risk factors for disseminated histoplasmosis are HIV infection, immunosuppressive disorders, immunosuppressive medications such as glucocorticoids, antirejection medications for organ transplant recipients, or tumor necrosis factor (TNF)-alpha inhibitor therapies. A separate group of patients, most of whom are middle-aged to older men, have chronic progressive disseminated histoplasmosis and have no known underlying immunosuppression. Our immunocompetent patient had reactivation of disseminated histoplasmosis with stroke-like symptoms.
